# Increased intestinal-fatty acid binding protein in obesity-associated type 2 diabetes mellitus

**DOI:** 10.1371/journal.pone.0279915

**Published:** 2023-01-26

**Authors:** Dicky L. Tahapary, Atikah I. Fatya, Farid Kurniawan, Cicilia Marcella, Ikhwan Rinaldi, Tri J. E. Tarigan, Dante S. Harbuwono, Em Yunir, Pradana Soewondo, Dyah Purnamasari

**Affiliations:** 1 Division of Endocrinology, Metabolism, and Diabetes, Dep artment of Internal Medicine, Faculty of Medicine Universitas Indonesia, Depok City, Indonesia; 2 Metabolic, Cardiovascular, and Aging Research Cluster, The Indonesian Medical Education and Research Institute, Faculty of Medicine, Universitas Indonesia, Jakarta, Indonesia; 3 Department of Internal Medicine, Fa culty of Medicine Universitas Indonesia, Jakarta, Indonesia; 4 Division of Hematology and Medical Oncology, Depa rtment of Internal Medicine, Faculty of Medicine Universitas Indonesia, Jakarta, Indonesia; 5 Clinical Epidemiology and Evidence-based Medicine Unit, Faculty of Medicine, Universitas Indonesia, Jakarta, Indonesia; Istanbul Bakirkoy Prof Dr Mazhar Osman Ruh Sagligi ve Sinir Hastaliklari Egitim ve Arastirma Hastanesi, TURKEY

## Abstract

**Background:**

Obesity is a traditional risk factor for type 2 diabetes mellitus (T2DM). However, recent studies reported that metabolically unhealthy obesity (MUO) exerts a higher risk of developing T2DM than metabolically healthy obesity (MHO) because of its higher state of insulin resistance. This may happen due to metabolic endotoxemia through gut dysbiosis and increased intestinal permeability. Our study aimed to know the association of intestinal permeability using intestinal fatty acid-binding protein (I-FABP) with obesity-related T2DM patients in Indonesia.

**Methods:**

This was a cross-sectional study that recruited 63 participants with obesity defined using body mass index (BMI) classification for the Asia-Pacific population (BMI ≥25 kg/m2). All participants were then grouped into T2DM and non-T2DM based on American Diabetes Association (ADA) diagnostic criteria. The I-FABP levels were measured using the enzyme-linked immunosorbent assay method.

**Results:**

The I-FABP level of T2DM group was higher compared to non-T2DM group, namely 2.82 (1.23) ng/mL vs. 1.78 (0.81) ng/mL (p<0.001; mean difference 1.033 with 95% CI 0.51–1.55). This difference was not attenuated even after adjustment for age. The fitted regression model using linear regression was: i-FABP = 1.787+1.034*(DM) (R^2^ = 18.20%, standardized ß = 0.442, p<0.001).

**Conclusions:**

This study underscores the association of intestinal permeability with T2DM in people with obesity and supports the evidence of the potential role of intestinal permeability in the pathogenesis of obesity-related T2DM.

## Introduction

The increasing prevalence of obesity is in line with the rise in obesity-related cardiometabolic complications, including type 2 diabetes mellitus (T2DM) [1]. However, not all people with obesity have a similar risk for T2DM. Around 10–20% of people belong to metabolically healthy obesity (MHO) with a lower relative risk of T2DM [2, 3]. Metabolically healthy obesity and metabolically unhealthy obesity (MUO) differ in fat distribution, ectopic fat deposition, inflammatory markers, adipocyte dysfunction and insulin resistance [2]. The discrepancy in insulin resistance between these two indicates other factors that influence the mechanism of insulin resistance in obesity. One of the potential factors influencing this mechanism is intestinal dysbiosis through increased intestinal permeability [4].

Increased intestinal permeability, which is directly correlated with intestinal dysbiosis, may trigger chronic inflammation and insulin resistance through metabolic endotoxemia [4, 5]. Animal studies have shown an association between intestinal permeability and insulin resistance in obesity; however, human studies have been inconsistent [6–8]. Increased intestinal fatty acid-binding protein (I-FABP) in plasma reflects enterocyte loss and is inversely proportional to the degree of intestinal villi atrophy [9, 10]. This marker is suitable for obesity, which has rapid enterocyte turnover and shortened intestinal villi [6]. It has also been reported as an intestinal permeability marker in inflammatory and metabolic diseases, including T2DM [11–15]. Nonetheless, the published paper regarding its use in obesity-related T2DM is still rare and limited to Caucasians only [8]. Increased I-FABP level in people with obesity who exerted chronic hyperglycemia has been reported. However, their study only included severely obese patients who were candidates for bariatric surgery (body mass index [BMI] >40 kg/m^2^). They also used hemoglobin A1c [HbA1c] >6% as a cut-off for chronic hyperglycemia, which was in the range for prediabetes, despite some of the patients having long-standing diabetes and using medication [8].

To our knowledge, the available data were limited to Caucasians. Thus, this is the first study that investigated intestinal permeability in people with a lower degree of obesity in relation to their diabetes status in Asian population. Asians and Caucasians differ in terms of defining obesity according to the BMI cut-off since Asians have a higher body fat percentage and more visceral fat despite having relatively similar BMI [16, 17]. This study aims to investigate the association of I-FABP level, as an intestinal permeability marker, with obesity-related T2DM in Indonesia.

## Materials and methods

### Ethics statement

This research has been approved by the Health Research Ethics Committee, Faculty of Medicine Universitas Indonesia-Dr. Cipto Mangunkusumo Hospital (FMUI-RSCM) no. KET-480/UN2.F1/ETIK/PPM.00.02/2021, which was granted on 19^th^ May 2021. All patients provided their written informed consent.

### Participants

This was a cross-sectional study using secondary data from our larger study on gut microbiota profile in various spectrum of dysglycemia patients that has not been published yet. The study recruited participants with obesity based on BMI for Asia-Pacific population (BMI ≥25 kg/m2) aged 18 to 60 years old in FMUI-RSCM from July 2018 to August 2019. The medical history taken from interview and questionnaires as well as measurements from initial visit were used as baseline data. Participants with chronic gastrointestinal disorders, severe kidney and liver disorders, autoimmune diseases, history of taking steroids, non-steroidal anti-inflammatory drugs (NSAIDs) and antibiotics in the past month, pregnant or breastfeeding, were excluded. Participants included in this study underwent laboratory tests which were further grouped into T2DM and non-T2DM. Patients’ comorbidities such as hypertension and dyslipidemia were also evaluated.

### Anthropometric and biochemical measurements

Data used in the present study were extracted from our larger study. As in the larger study, all participants enrolled were measured for anthropometric parameters including weight (kg), height (cm), waist circumference (cm), hip circumference (cm) and waist-hip ratio (WHR) by standardized methods. Systolic and diastolic blood pressure was measured twice using a validated device and the measurements’ average was calculated. Blood samples were drawn after an overnight fasting and kept in the freezer with a temperature of -80°C. Biochemical profiles being measured were fasting plasma glucose (FPG), HbA1c, fasting insulin, total cholesterol (TC), high-density lipoprotein cholesterol (HDL-C), low-density lipoprotein cholesterol (LDL-C), and triglyceride (TG).

The serum level of I-FABP was measured using the enzyme-linked immunosorbent assay (ELISA) method (R&D systems-DY3078 DuoSet ELISA for FABP2) at the Metabolic, Cardiovascular and Aging Cluster, The Indonesian Medical Education and Research Institute, Faculty of Medicine Universitas Indonesia (IMERI-FMUI). The assay ranged from 31.2 to 2,000 pg/mL.

### BMI classification

Body mass index is calculated as weight (kilograms) divided by the square of the height (meters^2^) [18]. According to Asia-Pacific cut-off points, [16] as also recommended by national clinical guideline on type 2 diabetes mellitus management in Indonesian adults by The Indonesian Society of Endocrinology (PERKENI), [19] the BMI classification is as follow: underweight (<18.5 kg/m^2^), normal weight (18.5–22.9 kg/m^2^), overweight (23–24.9 kg/m^2^), obese (≥25 kg/m^2^). Obese groups are further categorized into obese I (25–29.9 kg/m^2^), obese II (30–34.9 kg/m^2^) and obese III/morbid obese ((≥35 kg/m^2^).

### Type 2 diabetes mellitus grouping

Participants were grouped according to American Diabetes Association (ADA) criteria for diabetes: T2DM (FPG ≥126 mg/dL and/or HbA1c ≥6.5% and/or history of DM/diabetes treatment) or non-T2DM (FPG <126 mg/dL and HbA1c <6.5% and no history of DM/diabetes treatment) [19, 20].

### Hypertension and dyslipidemia definition

Hypertension was defined as systolic blood pressure ≥140 mmHg and/or diastolic blood pressure ≥90 mmHg or on antihypertensive medication. Whereas, dyslipidemia was defined as abnormal lipid metabolism indicated by the levels of TC ≥200 mg/dL, LDL-C ≥100 mg/dL, HDL-C <40 mg/dL and/or TG ≥150 mg/dL or on lipid-lowering treatment.

### Statistical analysis

The data were analyzed from May to June 2021 and the statistical analysis was performed using Statistical Package for the Social Sciences (SPSS) IBM version 28.0. Numerical data were presented as mean ± standard deviation (SD) if normally distributed or as median (minimum-maximum) if not normally distributed. The mean difference of I-FABP level between two groups was analyzed using linear regression, which was further adjusted for confounding. The correlation between I-FABP and HbA1c levels was analyzed using Spearman correlation. A comparison of I-FABP level between subjects who consumed and did not consume lipid-lowering therapy was analyzed using independent T-test. A p-value of <0.05 was considered statistically significant.

## Results

We recruited 63 subjects with obesity, of which 34 subjects had T2DM. The majority of subjects were women (82.53%), aged >45 years (63.50%), obesity grade I (54.00%) and central obesity (93.70%). The T2DM group were older (p<0.001), had more hypertension (p = 0.049), lower LDL cholesterol (p = 0.013), and as predicted, higher FPG (p<0.001) and HbA1c (p<0.001) levels. (**Table 1**) In this group, 2 subjects were newly diagnosed with T2DM and had no history of antidiabetic therapy, while 70.58% (24/34) of subjects had been diagnosed with T2DM in less than 5 years and 17.65% (6/34) had T2DM for more than 10 years. Most subjects did not have any history of diabetic complications. Among them, 14 patients consumed only 1 oral anti-diabetic drugs (OAD), 12 subjects on dual OADs therapy, 2 subjects had combination of OADs and insulin therapy and 2 others did not take anti-diabetic medication.

**Table 1:** Subject characteristics.

Variable	T2DM	Non-T2DM	P value
	(n = 34)	(n = 29)	
Age (year)	50.65 ± 6.32	41.86 ± 11.16	**<0.001** ^**a**^
• >45 years old (%)	76.50	48.30	**0.021** ^**b**^
Female (%)	85.30	79.30	
BMI (kg/m^2^)	29.80 (25.11–42.50)	29.84 (25.73–39.10)	0.478^c^
Obesity			0.228^**b**^
• Grade I (%)	55.90	51.70	
• Grade II (%)	26.50	41.40	
• Grade III (%)	7.60	6.90	
Waist (cm)	94.53 ± 10.68	93.16 ± 10.43	0.612^**a**^
WHR	0.88 (0.80–1.03)	0.88 (0.81–1.01)	0.815^c^
Central obesity (%)	97.10	89.70	0.250^**b**^
Dyslipidemia (%)	52.90^#^	31.00^$^	0.08^**b**^
Hypertension (%)	44.10*	20.70^&^	**0.049** ^**b**^
FPG (mg/dL)	130.00 (83.00–311.00)	89.00 (75.00–105.00)	**<0.001** ^c^
HbA1c (%)	7.50 (4.70–13.20)	5.70 (4.70–6.40)	**<0.001** ^c^
Fasting insulin (mIU/L)	11.06 ± 5.88	9.58 ± 4.11	0.262^**a**^
I-FABP (ng/mL)	2.82 ± 1.23	1.78 ± 0.81	**<0.001** ^**a**^

Data presented as mean ± SD, median (min-max), or proportion (%).

T2DM: type 2 diabetes mellitus, BMI: body mass index, WHR: waist-to-hip ration, FPG: fasting plasma glucose, HbA1c: hemoglobin A1c.

^a^: linear regression

^b^: Chi-square test

^c^: Mann-whitney test.

^#^: 29.4% subjects were on lipid-lowering therapy in T2DM group

^$^:13.8% subjects were on lipid-lowering therapy in Non-T2DM group

*: 26.5% subjects were on antihypertensive therapy in T2DM group

^&:^ 17.2% subjects were on antihypertensive therapy in Non-T2DM group

**Fig 1:**
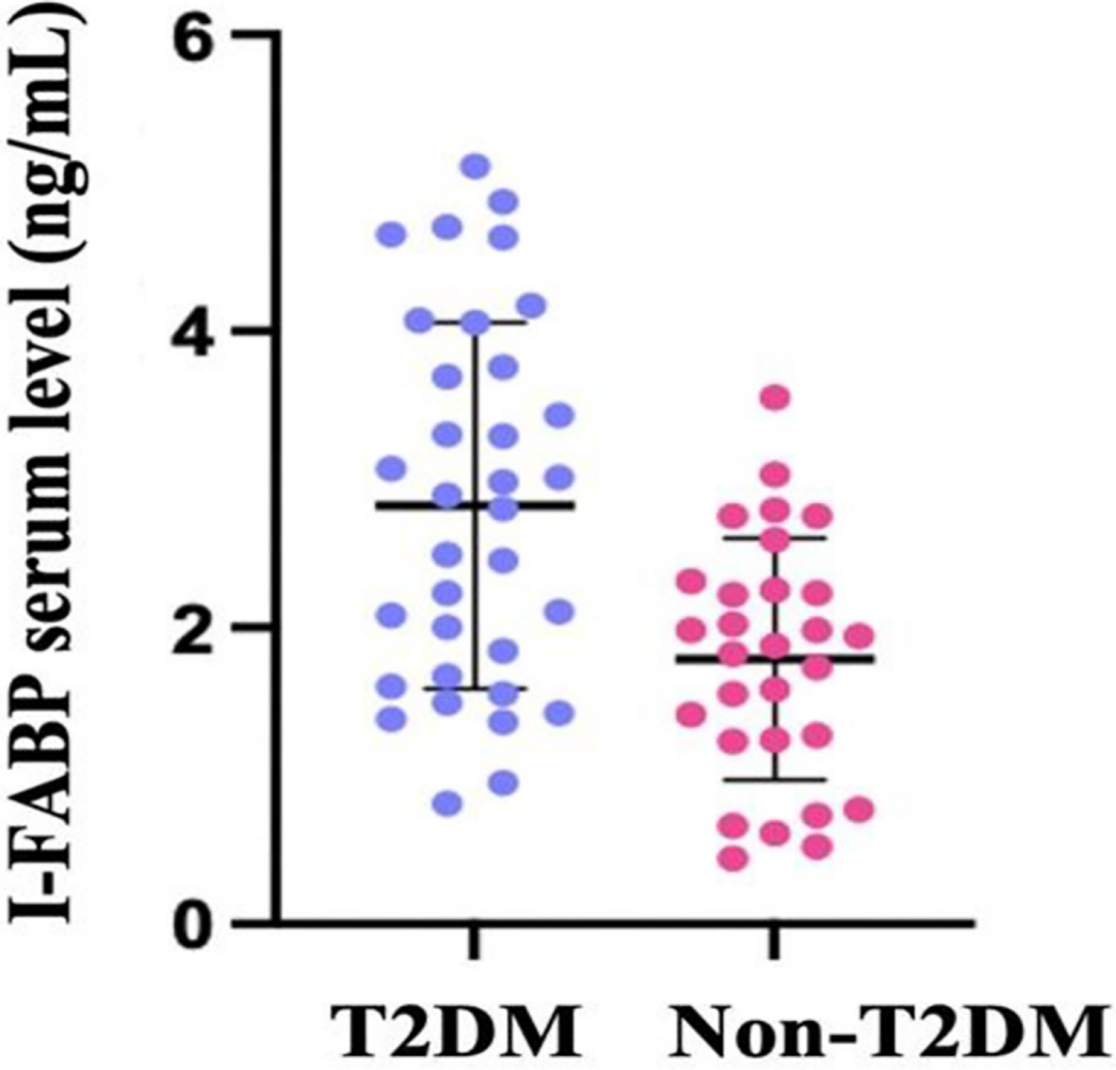
Serum level of I-FABP in T2DM and non-T2DM groups, were presented in dot-plot form with mean (SD).

The mean I-FABP level in the group with T2DM was higher, namely 2.82 (1.23) ng/mL vs. 1.78 (0.81) ng/mL (p<0.001; mean difference 1.033 with 95% CI 0.51–1.55). (**Fig 1**) We analyzed the mean difference between two groups using linear regression and the fitted regression model was: i-FABP = 1.787+1.034*(DM) (R^2^ = 18.20%, standardized ß = 0.442, p<0.001). Other confounding factors that might interfere the result such as age and sex were analyzed. This difference was still significant even after adjustment for age difference. The fitted regression model after adjustment for age was: i-FABP = 0.470+0.757*(DM)+0.031*(age). The overall regression was statistically significant (R^2^ = 22.6%, standardized ß = 0.324, p = 0.012).

We also analyzed the correlation between I-FABP and HbA1c levels using Spearman correlation to evaluate the relationship of gut permeability with hyperglycemia. The I-FABP level was positively correlated with the HbA1c level, however the association was weak (r = 0.251, p = 0.047). To see if there was an effect of lipid-lowering therapy on I-FABP level, we compared the I-FABP levels between subjects who consumed lipid-lowering therapy and those who did not. There was no significant difference found between those groups (p = 0.608).

## Discussion

This study observed a higher I-FABP level among participants with obesity and T2DM in comparison to those without T2DM. This result supports the evidence on the potential role of intestinal permeability in the pathogenesis of T2DM in participants with obesity [8].

Our study supports previous studies in other countries that reported increased I-FABP, as gut integrity marker, in T2DM [12–15]. These findings suggest that increased intestinal permeability due to intestinal dysbiosis is associated with systemic inflammation and insulin resistance, [6, 12, 21] potentially leading to T2DM. It has been reported that increased lipopolysaccharide (LPS) level in T2DM patients was associated with pro-inflammatory cytokines [22]. There was an increase in C-reactive protein (CRP), interleukin-6 (IL-6), tumor necrosis factor-alpha (TNF-α) and a predominant T helper 1 (Th 1) pathway [13, 21, 22]. It has also been reported that the association of I-FABP levels with CRP, blood glucose and TG levels were still significant even after adjustment for age and BMI [15]. The impairment of intestinal integrity might provide the influx of LPS to the systemic circulation and induce metabolic endotoxemia, resulting in low-grade systemic inflammation and consequently, insulin resistance.

Interestingly, this study mean I-FABP serum level was higher than in previous studies. This might be due to the fact that all subjects in this study had obesity, and most of them also had abdominal obesity. Furthermore, this might also be influenced by the differences in genetics, race and geographical location, which contributed to different enterotypes, and types of physical activity and diet, thus resulting in the differences in intestinal permeability. Intestinal permeability is closely associated with intestinal microbiota composition [23].

Geographical location determines the dominant enterotype, where healthy Indonesians belong to enterotype II, which is dominated by the genus Prevotella. This finding is different from the dominant enterotype in other Asian countries such as Japan, China, and Taiwan, which belongs to enterotype I, and western countries which are more abundant in Bacteroides (enterotype III) [24]. Westernized diet, which commonly comprises high-fat, high-carbohydrate, and low-fiber, is correlated with increases in Firmicutes and Proteobacteria. In contrast, an increase in the Firmicutes/Bacteroidetes ratio is associated with higher BMI, intestinal permeability, and insulin resistance [6, 25].

The frequency of physical activity also affects microbiota composition. Several studies reported that daily physical activity increased the diversity of Firmicutes bacteria, which then improves intestinal permeability by producing tight-junction protein [23]. In this study and previous studies, there was no analysis of the diet and activity patterns of the subjects. However, the results of this study were in line with changes in dietary patterns and lifestyles in Indonesia, especially in urban areas, which adopt a Westernized diet and a sedentary lifestyle, thereby potentially changing the composition of the gut microbiota and increasing intestinal permeability in people with obesity.

Other factors that contribute to variations in intestinal permeability are age and sex [23]. Intestinal dysbiosis is higher in older age, which is in line with the results of research by Rahayu et al who reported a reduced gut microbiota diversity and an increase in the proportion of Enterobacteriaceae, Coliform and Escherichia coli in the elderly population in Indonesia [26]. From multivariate analysis, the I-FABP levels were independently associated with either T2DM or age. This is consistent with the previous study in which I-FABP levels were associated with T2DM, even after controlling the confounding variables like age and BMI [12].

Intestinal-FABP is specifically synthesized in enterocytes and released to systemic circulation whenever enterocyte apoptosis/necrosis occurs [10]. Increased I-FABP serum levels indicate greater gut integrity loss in T2DM patients. This is in line with some intestinal mucosal changes in obesity and T2DM, including increased small intestinal enterocyte mass as well as the length of crypt and villi due to faster enterocyte turnover [6, 15]. However, I-FABP serum levels were correlated with duodenal *FABP2* gene expression thus, there was a possibility that high I-FABP serum levels were due to its increased pool production rather than increased enterocyte loss [15]. It has been previously postulated that the I-FABP serum levels increase in people with morbid obesity and chronic hyperglycemia representing higher enterocyte loss through the role of *FABP2* gene expression [8]. Meanwhile, others have reported that the production of I-FABP was determined partly by duodenal *FABP2* gene expression, but its serum concentration was affected by BMI and insulin resistance. It is suggested that there was an increase in enterocyte loss in the presence of insulin resistance, particularly in uncontrolled diabetes [15]. In contrast to the previous study, [27] our study observed a weak positive correlation between I-FABP and HbA1c levels, which supports the potential effect of uncontrolled diabetes on enterocyte loss. In the opposite direction, our finding also aligns with the hypothesis that metabolic endotoxemia triggers insulin resistance and hyperglycemia through the disrupted intestinal barrier [4]. Another interesting view is that long-standing hyperglycemia leads to disrupted gut epithelial integrity [28]. Therefore, further evaluation should be addressed to evaluate the causal relationship between those two.

Despite being the first study to assess the association of I-FABP level, as an intestinal permeability marker, with obesity-related T2DM in Indonesia, there were some limitations in the study. Firstly, this is a cross-sectional study, thus no causal inference can be generated. Secondly, it did not analyze the effect of diet, physical activity and antidiabetic drugs. We also did not assess the inflammation markers, as previous studies have showed increased I-FABP level was related to systemic inflammation. Furthermore, as most participants were women, whether this association found in men needs further study.

In conclusion, the findings of this study support an association between intestinal permeability and obesity-related T2DM, which highlights the involvement of the intestinal barrier in the pathophysiology of T2DM in obesity. This indicates the potential role of using one of the intestinal permeability markers to predict the risk of developing T2DM in obesity in the future and is likely to help classifying them as MHO and MUO. Furthermore, improving the intestinal barrier is promising to be one of the treatment strategies to prevent obesity-related complications.

## Supporting information

S1 Data (XLSX)
